# Multifocal Electroretinography in Assessment of Macular Function after Internal Limiting Membrane Peeling in Macular Hole Surgery

**DOI:** 10.1155/2019/1939523

**Published:** 2019-03-27

**Authors:** M. Y. Faria, D. C. Sousa, S. Mano, R. Marques, N. P. Ferreira, A. Fonseca

**Affiliations:** ^1^Ophthalmology University Clinic, Faculdade de Medicina Lisboa, Universidade de Lisboa, Lisbon, Portugal; ^2^Ophthalmology Department, Hospital Santa Maria, Centro Hospitalar Universitário Lisboa Norte, Lisbon, Portugal

## Abstract

**Purpose:**

Internal limiting membrane (ILM) peeling is important for macular hole (MH) surgery but may have secondary effects visible on spectral domain optical coherence tomography (OCT) and multifocal electroretinography (mfERG). We relate integrity of inner and outer macular layers with functional results with mfERG.

**Methods:**

Nonrandomized prospective study of 33 consecutive eyes of 33 patients with macular hole who underwent vitrectomy with ILM peeling. Best-corrected visual acuity was assessed, and integrity of external layers was measured using OCT. Each component of mfERG, N1 and P1 amplitude and latency, was also measured.

**Results:**

All eyes showed macular hole closure. Visual acuity improved from 20/400 to 20/40 in the Snellen visual acuity chart (*P* < 0.001), and OCT external lines were intact in 19 eyes and disrupted in 14 eyes. Postoperatively, N1 and P1 amplitudes in ring 1 increased compared to preoperative values (*P* < 0.001 for both). Latency remained delayed for both N1 and P1 wave. In the group of 19 eyes with integrity of outer retinal layers, N1 amplitude in ring 1 was superior to the group of 14 patients with disrupted outer retinal layers (*P*=0.042).

**Conclusions:**

In macular hole surgery, structure analysis in OCT is one of the important outcomes for the retinal surgeon. Functional results are parallel with anatomic results, but visual gain is limited. The limited recovery in mfERG suggests an alteration of retinal physiology that could explain limited vision recover.

## 1. Introduction

Currently, macular hole is a common surgical feature, practiced in most vitreoretinal centers with high rate of closure [1]. Peeling of the internal limiting membrane (ILM) and inverted flap technique allows this high closure rate [2]. Indications for surgery and surgical technique to be used are based on structural images. Also, postoperative results are based on foveal anatomy. With ocular coherence tomography (OCT), it has become much easier to define and understand the retinal anatomy before and after macular hole surgery. In addition, it has also helped to explain poor visual acuity (VA) in cases where the retina appeared normal at biomicroscopic examination. Most efforts have focused on surgical technique to realign photoreceptors and improve VA. However, exploring retinal function is another way of understanding the healing process of macular holes.

After successful hole closure, patients refer less metamorphopsia and an improved ability to read [3]. Nevertheless, even with hole closure, visual function improves but the gain in visual acuity is often limited. Visual function before and after macular hole surgery is usually assessed by visual acuity measurement [4]. However, the visual acuity level represents only a part of the visual function resulting from macular hole development, which includes metamorphopsia, scotoma, and blurred vision [5].

Because ILM is the basement membrane of Müller cells, the inner barrier of the neural retina and anatomically adjacent to the retinal nerve fiber layer (RNFL) and ganglion cell layer (GCL), ILM removal may alter the architecture of the inner retinal layer and also alter the function of Müller cells, responsible for the generation of electroretinogram [6]. Nerve fibers are joined and fixed at the lamina crivosa, and after ILM peeling, a dissociated optic nerve layer has been referred by Tadayoni et al. [7]. Also, nasal displacement and contraction of this neural layer and ganglion cell axon allow the underlying macular tissue to react with the alteration of cytoarchitecture of external retinal layers [8]. All these structural alterations may have consequences on postoperative macular function. Moreover, the direct trauma of surgery to the nerve fiber layer, the gas tamponade, the stain used to visualize the transparent ILM, and the endoillumination light probe are all factors to consider as possible toxic effects to macular function.

Multifocal electroretinography (mfERG) is an objective clinical tool to assess visual function and selects the multiple retinal locations of the macular area to provide a topographic map of local central retinal electrophysiological activity [9]. The purpose of this prospective study was to characterize macular function by means of mfERG, before and after surgery.

## 2. Methods

### 2.1. Setting and Patients

In a nonrandomized prospective study, 33 consecutive eyes of 33 patients with MH underwent surgery between January 2015 and June 2017 at the Department of Ophthalmology of Santa Maria Hospital, Lisbon. Exclusion criteria were maculopathy other than MH, surgeries for MH recurrence, other retinal diseases, or an axial length greater than 26.0 mm. Mean follow-up time was at least 12 months after surgery. This study was approved by the Ethics Committee of Santa Maria Hospital. The tenets of the Declaration of Helsinki were followed. All subjects have given written informed consent to the surgical and the study procedures.

### 2.2. Collected Data

Preoperative data included age, gender, and complete ophthalmic examination. Best-corrected visual acuity (BCVA) was measured using a Snellen chart and converted to the logarithm of the minimum angle of resolution (logMAR) for statistical analysis. Hand motion was considered as logMAR 3 and counting fingers as logMAR 2.

### 2.3. Optical Coherence Tomography

All macular holes were staged based on recent OCT-based classification [10], and only full thickness macular hole, grade 2 to 4, was considered for the study. Retinal images were acquired using Spectralis SD-OCT (Heidelberg Engineering, Heidelberg®, Germany), using the eye-tracking feature with software posterior pole images centered on the fovea (61 acquisitions, 120 *μ*m interval).

**Figure 1:**
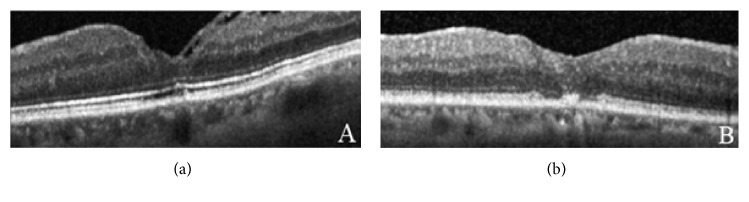
OCT with closed macular hole: integrity of ELM and EZ zone (a) and disrupt ELM and EZ (b).

The status of the foveal ellipsoid zone (EZ) and the external limiting membrane (ELM) were examined for each eye to test integrity: intact and disrupted. The intact eyes had a regular continuation of the hyperreflective line corresponding to the EZ or ELM. The disrupted eyes were characterized by hyporeflective discontinuities in the EZ or ELM line (Figure 1). These classifications were assessed by agreement of two authors (MF and NF).

### 2.4. Multifocal Electroretinography

RETIscan Multifocal ERG (Version 6.12.5.12; Roland Consult) was used for mfERG recording. The recording procedures were the same as those described by the International Society for Clinical Electrophysiology of Vision [11]. The stimulus consisted of 61 hexagons that scale concentrically and covered the central 25 degrees of the fundus area. The viewing distance was 29 cm, which allowed a viewing angle of approximately 30 degrees. Each hexagon was modulated temporally between black (2 cd/m^2^) and white (200 cd/m^2^). Pupils were dilated with tropicamide and phenylephrine hydrochloride. After topical anesthesia, a contact lens jet electrode was placed, and signals were recorded. During the recordings, the patients' fixations were monitored. The signal was amplified (100,000) and bandpass filtered (10–300 Hz). Three-dimensional topography represents the retinal response density (amplitude per retinal area, nV/deg^2^).

**Figure 2:**
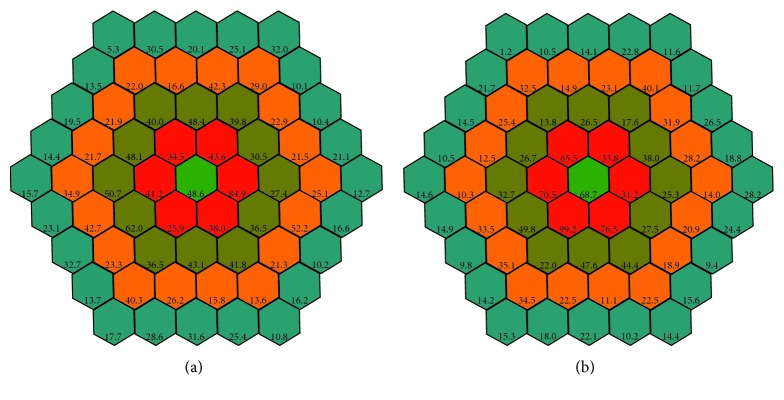
Amplitude of P1 nV/deg^2^ in patient no. 4 in five rings centered in fovea, before surgery (a) and after surgery (b) Ring 1 (green) and ring 2 (red) are considered for study.

**Figure 3:**
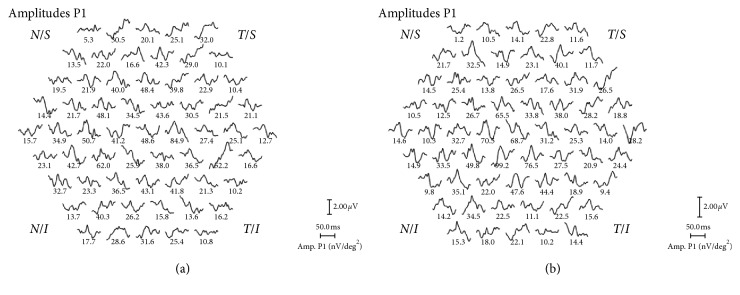
Amplitude of P1 in nV/deg^2^ in patient no. 4 in topographic display around fovea, before (a) and after (b) surgery.

**Figure 4:**
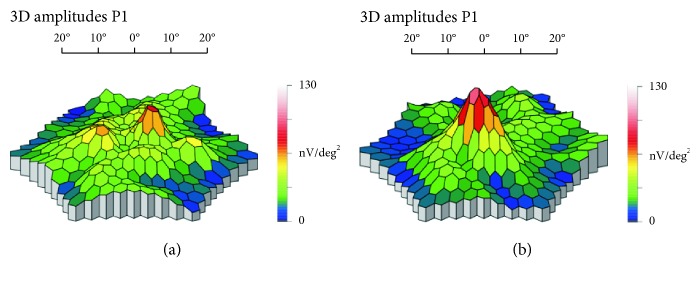
Three-dimensional topography of amplitude P1 of patient no. 4, before and after surgery for macular hole.

The mean simultaneous response was recorded. The typical waveform of the basic mfERG response is a biphasic wave with an initial negative deflection followed by a positive peak. Implicit times (latencies) and the amplitude relative to their respective areas (nV/deg^2^) of the first negative peak (N1) and the first positive peak (P1) were measured using regional averages derived from 5 concentric rings (Figures 2 and 3). Three-dimensional topography (Figure 4) represents the retinal response density (amplitude per retinal area, nV/deg^2^). The studied field contained 61 hexagons in 5 rings within a field diameter of 25 degrees, 12.5 degrees radially centered on the fovea [12], and was analyzed with RETIscan software. Five rings correspond to 5 degree areas. Only ring 1 and ring 2 were considered, as they roughly parallel a 3 mm diameter ILM peel during surgery.

Multifocal ERG was recorded preoperatively and at 12 months after surgery. In the present study, we focused on amplitude and latency of N1 and P1, before and after surgery.

### 2.5. Surgical Procedure

A standard surgical procedure consisted of 23- or 25-gauge, three-port pars plana vitrectomy (PPV). Except in pseudophakic patients, every patient underwent combined cataract surgery, in order to avoid confounding results. Standard small-incision phacoemulsification and implantation of a standard foldable intraocular lens were associated with vitrectomy. In every eye, Brilliant Peel® Dual (Geuder, Germany) assisted ILM peeling was performed, in an area of approximately 3 mm, engaged with end-grip intraocular forceps. A flap is created, then peeled in a rosette way all around the hole, and trimmed until the border of the macula but leaving a flap big enough to invert and cover the hole. BSS was exchanged with 15% SF6 (sulfur hexafluoride) gas. The patients were instructed to maintain a face-down position for at least 5 days. All surgeries were performed by the same experienced surgeon (MF).

### 2.6. Postoperative Follow-Up

In postoperative observations, at day 1, month 3 and month 6, a thorough ophthalmic examination, including BCVA, mfERG, and OCT, whenever possible, and fundus revision were performed. Hole status, ELM, and EZ integrity at the fovea were measured in OCT. N1 and P1 waves of mfERG were measured for amplitude and implicit time.

### 2.7. Statistical Analysis

The results are expressed as medians (range). Only ring 1 and ring 2 of mfERG were considered as they correspond to the area of the ILM peel. Amplitude and latency of N1 and P1 of mfERG were compared before and after surgery. BCVA and amplitude of N1 and P1 of mfERG were compared between the groups with intact or disrupted photoreceptor. For comparisons before and after surgery, the Wilcoxon signed-rank test was used. Between group analyses were performed with the Mann–Whitney *U* test. Correlations were tested using Spearman's *ρ* correlation coefficient. Statistical significance was established at *P* < 0.05.

## 3. Results

### 3.1. Demographic and Clinical Data

The median (range) age of the patients was 71 (21) years, and the study group included 14 men and 19 women. Thirty-three eyes were studied, 26 patients underwent concomitant cataract surgery, and 7 were already pseudophakic. None of the patients required further treatment during the 12-month follow-up.

### 3.2. Amplitude of P1 and N1 and Respective Latency Times before and after Surgery

There was an increase in amplitude of P1 and N1 waves, in ring 1, after surgery (*P* < 0.001 for both). Ring 2 increase was only statistically significant for P1 wave (*P*=0.040). Pre- and postoperative latency was not significant for neither waves (Table 1).

### 3.3. Visual Acuity and Amplitude of P1 and N1 by Photoreceptor Status before and after Surgery

The median (range) visual acuity, in logMAR, improved from 2.10 (2.90) to 0.70 (4.80), *P*=0.007 and from 1.80 (1.60) to 1.10 (2.0), *P*=0.008, from baseline to 12 months in the intact and disrupted photoreceptor groups, respectively. P1 and N1 in the first ring region of the retina increased both in the intact photoreceptor group and the disrupted photoreceptor group (*P* < 0.001 and *P*=0.001, respectively), with no differences in the second-ring regions of the retina (Table 2).

### 3.4. Difference between Post- and Presurgery in BCVA, N1 Amplitude, and P1 Amplitude

Median (range) increase in visual acuity from pre to postsurgery was −0.80 (7.60) logMAR and −0.40 (1.40) logMAR, in the intact photoreceptor group and the disrupted photoreceptor group, respectively (*P*=0.114). Increase in N1 ring 1 was 30.20 (75.20) nV/deg^2^ and 10.25 (116.90) nV/deg^2^, and increase in P1 ring 1 was 12.00 (99.10) nV/deg^2^ and 20.95 (97.90) nV/deg^2^ in the intact photoreceptor group and the disrupted photoreceptor group, respectively (*P*=0.042 and *P*=0.418, respectively), as assessed by the Mann–Whitney *U* test. There was no correlation between BCVA increase and N1 increase in any of the groups, intact or interrupted, as assessed by Spearman's *ρ* correlation coefficient.

## 4. Discussion

In macular hole surgery, removing the ILM may eliminate almost all traction, anterior-posterior and tangential, and lead to a higher probability of hole closure [13]. ILM peeling was introduced as an additional maneuver to improve anatomical and functional outcomes [14], allowing 100% closure of idiopathic macular holes, especially with the inverted flap technique [2]. With the latest spectral domain OCT, with increased depth of resolution, it has become much easier to define and understand the retinal anatomy before and after macular hole surgery. In our study, peeling was performed in every surgery. However, the effects of ILM removal on retinal function remain unknown. Multifocal electroretinography is a noninvasive method that objectively measures visual function by selecting multiple retinal locations around macular area to provide a topographic map of electrophysiological activity in the central retina [9]. Based on the International Society for Clinical Electrophysiology of Vision (ISCEV), mfERG responses show greater amplitudes in the fovea where cone photoreceptors and bipolar cells are in greater number. It is believed that N1 is generated by photoreceptors in the outer retinal layer and P1 is generated by Müller and bipolar cells [15, 16].

If outer retinal layers are intact after surgery, photoreceptors will probably recover function, N1 will probably increase and influence internal layers, in recovering P1 functions. However, that is not always true, as peeling of ILM may negatively influence these internal layers.

Several previous studies with mfERG on eyes before and after MH surgery were published. Machida et al. [17] found no toxic effect of brilliant blue *G*, indocyanine green, or triamcinolone acetonide in macular hole surgery with ILM peel, as measured with focal macular electroretinograms. Scupola et al. [18] compared triamcinolone acetonide and infracyanine green in thirty eyes studied with focal electroretinogram and found late toxic effect with infracyanine green-assisted ILM peeling. Bellerive et al. [19] also compared toxicity of trypan blue and infracyanide green in macular hole surgery using mfERG before and after surgery and concluded that, at 12 months, there was improvement of P1 amplitude and implicit time, BCVA, and contrast sensitivity was not different between groups. Ferencz et al. [20] studied mfERG in 30 eyes with MH, found preoperative subnormal responses, and only at 20 months found significant improvement in both groups by probable toxicity with infracyanine green in surgery.

Studying macular hole before and after its closure is a unique situation where outer retinal cells do not exist at full thickness hole. After successful surgery, there is a closed hole with or without integrity of ELM, EZ, and retinal pigment epithelium. Also, there is a reduced thickness of internal macular layers after ILM peeling in macular hole surgery [8], especially ganglion cell layers and internal nuclear layers, measured at 3 mm diameter centered on fovea, roughly the degree reached by the second ring in mfERG.

In this macular hole study, mfERG recorded before surgery showed almost undetectable retinal response in foveal and parafoveal areas, in ring 1 and ring 2. After surgery, the improvement in the retinal response density of mfERG in the same ring seems to be consequent to closure of the macular hole with realignment of photoreceptor cells and glial cell activation. Resolution of the central scotoma seems to be attributed to anatomical repair and, in our study, we found a statistically significant increase in N1 and P1 in ring 1. Comparing ring 1 in N1 and P1 amplitude and outer retinal layer status, we found that P1 wave increased both in the intact and disrupted groups (*P* < 0.001 and *P*=0.001, respectively), which is consistent with increase of P1 amplitude with closure of hole, whatever the outer layer status is. As to the N1 wave, the increase in the intact group was superior to the increase in the disrupted group (*P*=0.042). All other results were not significant. Therefore, in our results, correct restoration with intact hyperreflective photoreceptor lines in OCT, ELM and EZ, results in improvement in outer retina response, as measured by the N1 wave. It has generally been thought that photoreceptor status by OCT is associated with visual recovery [21]. Although the N1 amplitude was reduced by the presence of MH, the increase in 66% of its amplitude after ILM peeling, hole closure, and realignment of photoreceptors agrees well with that the N1 amplitude was related to and generated from the outer retina.

The median BCVA of patients included in the study improved after surgery. The mechanism by which visual function improves after surgery is not clearly understood. However, the centripetal movement of the previously displaced photoreceptors to its original site as proven by OCT images may be the simplest explanation [4]. However, even with integrity in photoreceptor lines, vision improvement is limited.

BCVA increased in both the intact and disrupted groups (*P*=0.007 and *P*=0.008, respectively). Even though clinically the results are better in the intact group, there is a large variability, which may have prevented a higher statistical significance in this group.

Besides the low recovery of P1, there was also a delay of implicit time. After ILM peeling and closure of the macular hole, implicit time of the P1 wave in ring 1 maintained delay of 33.96 (38.20) millisecond in this study compared to normative data [22]. The delay may be related to surgical aggression of ILM peeling, or even ischemia in the macular area [23]. Implicit time was delayed before surgery and never recovered, even after closure and visual recovery. Andréasson and Ghosh already referred [24] to a very slow cone function recovery even after successful anatomical healing.

Hood et al. [9] already suggested that the multifocal ERG value might affect numerous factors we could not detect and control. As to the N1 wave, the increase in the intact group was superior to the increase in the disrupted group (*P*=0.042), and therefore, the P1 wave in the intact group was expected to increase superiorly than that in the disrupted group. However, P1 ring 1 increased both in the intact and disrupted groups, with no statistical differences, suggesting a loss of interaction with outer retinal layers.

Thus, we suspect that ILM peeling may damage inner retinal layers and Muller cell function which have some negative effect on the P1 wave of multifocal ERG and visual acuity.

In eyes with successful MH surgery, there is disappearance of central scotoma and improvement of visual acuity. This improvement is enhanced if there is realignment of photoreceptors and integrity of outer retinal layers. This results in N1 increase in mfERG of 66% compared to 53% increase in the disrupt photoreceptor group.

P1 wave in mfERG also increases after surgery, 69% in intact and 109% in disrupted outer retinal layers, but below age-related normative data. This uneven increase of the P1 wave, whatever the outer retinal status is, may explain the functional impairment between inner and outer retinal layers. ILM is the end feet of glial cells, and an alteration of retinal physiology in inner retinal layers, where bipolar and glial cells interact with photoreceptors, may explain the limited improvement in BCVA.

The present study had several limitations. The number of patients included is small, which may limit the reliability of the statistical results. Also, only BCVA was measured, and no other visual functions were tested. We did not have a control group in which the ILM was not peeled in macular hole surgery. Performing the surgery without ILM peeling could lead to lower closure rates in grade 3 or 4 macular hole, and therefore, generating such a control group would be unethical. The peeled area of ILM is around 3 mm in diameter, but no film registration of each surgery was made in every patient to ensure the exact diameter of the peel.

Lastly, contrary to most diagnostic equipment in ophthalmology, normative data in mfERG may vary with each equipment, and laboratory and healthy fellow eyes were not used as control. Also, population-specific factors such as age, ethnicity, pupil size, axial length, and diurnal variation may influence normative data.

## 5. Conclusion

In eyes with successful MH surgery, there is disappearance of central scotoma and improvement of visual acuity. This improvement is enhanced if there is realignment of photoreceptors and integrity of outer retinal layers. However, functional results may not be parallel with anatomic results as visual gain is limited. ILM is the end feet of glial cells, and an alteration of retinal physiology in inner retinal layers, where bipolar and glial cells interact with photoreceptors, may explain the limited improvement in BCVA. The limited recovery in mfERG suggests an alteration of retinal physiology that could explain limited vision recover.

## Figures and Tables

**Table 1:** Amplitude of P1 and N1 and respective latency times before and after surgery.

Parameter	Before surgery (*n*=33)	After surgery (*n*=33)	*P* value
P1 amplitude (nV/deg^2^)			
Ring 1	36.00 (77.20)	59.20 (92.60)	**<0.001**
Ring 2	45.70 (62.30)	55.30 (97.30)	**0.040**

P1 latency (ms)			
Ring 1	33.30 (30.40)	34.30 (38.20)	0.806
Ring 2	33.30 (20.60)	34.30 (30.40)	0.955

N1 amplitude (nV/deg^2^)			
Ring 1	28.30 (127.30)	46.30 (155.30)	**<0.001**
Ring 2	20.80 (85.80)	22.60 (47.40)	0.550

N1 latency (ms)			
Ring 1	17.60 (48.80)	18.60 (22.50)	0.330
Ring 2	17.60 (13.70)	17.60 (27.40)	0.977

Amplitude of P1 and N1 are expressed in nanovoltage (nV)/area degree^2^ (deg^2^) and implicit time (latency) in milliseconds (ms). All values are expressed as median (range). *P* values are obtained from the Wilcoxon signed-rank test.

**Table 2:** Visual acuity and amplitude of P1 and N1 by photoreceptor status before and after surgery.

Parameter	Before surgery (*n*=33)	After surgery (*n*=33)	*P* value
Intact photoreceptor			
Visual acuity (logMAR)	2.10 (2.90)	0.70 (4.80)	**0.007**
P1 amplitude (nV/deg^2^)			
Ring 1	37.70 (77.20)	59.00 (92.60)	**<0.001**
Ring 2	51.50 (39.70)	67.30 (97.30)	0.231
N1 amplitude (nV/deg^2^)			
Ring 1	31.40 (120.80)	67.90 (117.80)	**<0.001**
Ring 2	20.30 (30.80)	24.80 (47.40)	0.571

Disrupted photoreceptor			
Visual acuity (logMAR)	1.80 (1.60)	1.10 (2.00)	**0.008**
P1 amplitude (nV/deg^2^)			
Ring 1	35.65 (48.10)	60.80 (82.20)	**0.001**
Ring 2	35.15 (62.30)	45.85 (80.20)	0.084
N1 amplitude (nV/deg^2^)			
Ring 1	23.35 (127.30)	31.65 (155.30)	**0.001**
Ring 2	21.30 (85.80)	21.50 (40.20)	0.861

Amplitude of P1 and N1 are expressed in nanovoltage (nV)/area degree^2^ (deg^2^). All values are expressed as median (range). *P* values are obtained from the Wilcoxon signed-rank test.

## Data Availability

All data used to support the findings of this study are at Ophthalmology Department, Hospital Santa Maria, Centro Hospitalar Universitário Lisboa Norte, and available from the corresponding author upon request.
